# Patient Related Factors Affecting Adherence to Antimalarial Medication in an Urban Estate in Ghana

**DOI:** 10.1155/2015/452539

**Published:** 2015-02-12

**Authors:** Alexandria O. Amponsah, Helen Vosper, Afia F. A. Marfo

**Affiliations:** ^1^School of Pharmacy and Life Sciences, Robert Gordon University, Aberdeen AB10 7QG, UK; ^2^Oakalex Community Pharmacy, Accra, Ghana; ^3^Department of Clinical & Social Pharmacy, Faculty of Pharmacy, KNUST, Kumasi, Ghana

## Abstract

Our aim was to measure the adherence to Artemisinin based Combination Therapy and to determine patient related factors that affect adherence. Three hundred (300) patients receiving ACT treatment dispensed from the community pharmacy were randomly selected and followed up on the fourth day after the start of their three-day therapy to assess adherence. Adherence was measured by pill count. Quantitative interviews using a semistructured questionnaire were used to assess patients' knowledge and beliefs on malaria and its treatment. Adherence levels to the ACTs were 57.3%. Patient related factors that affected adherence to ACTs were patients' knowledge on the dosage (*P* = 0.007; *v* = 0.457), efficacy (*P* = 0.009; *v* = 0.377), and side effects (*P* = 0.000; *v* = 0.403) of the ACTs used for the management of malaria, patients' awareness of the consequences of not completing the doses of antimalarial dispensed (*P* = 0.001; *v* = 0.309), and patients' belief that “natural remedies are safer than medicines” and “prescribers place too much trust in medicines.” There was no significant relationship between adherence and patients' knowledge on the causes, signs, and symptoms of malaria. There is the need for pharmacy staff to stress on these variables when counseling patients on antimalarials as these affect adherence levels.

## 1. Introduction

Malaria is still a concern in most developing countries in Africa. Although, in 2010, malaria caused 660,000 deaths mostly among African countries, malaria mortality rates are gradually falling and have reduced by more than 33% in Africa [1]. In Ghana, about 32.5% of all outpatient attendance and 48.8% admissions of children under the age of 5 are due to malaria [2]. For decades, chloroquine (CQ) was used as first-line treatment for malaria in many developing countries, including Ghana. However, its use has become limited due to the emergence of resistance and subsequent treatment failure. Factors which may have contributed to chloroquine treatment failures include incorrect dosing, noncompliance with duration of the dosing regimen, poor drug quality, drug interaction, and misdiagnosis [3].

Over the past few years, a new group of antimalarials, the Artemisinin Based Combination Therapy (ACT) compounds, found to produce a very rapid therapeutic response have been introduced. These medicines are active against multidrug resistant* Plasmodium falciparum* [4]. In line with World Health Organization (WHO) recommendations, nearly all high-burden African countries including Ghana have moved from chloroquine to ACTs as first-line treatment. In 2008, there were reports at the Thai-Cambodian border that the ACTs were losing their potency [5]. This is further backed by a study in Parlin, western Cambodia, which indicates that some resistance has been developed towards the ACTs [6]. WHO also acknowledges the fact that rational uses of antimalarial medicines are critical; it suggests that resistance to the ACTs could unravel national malaria control programmes [7]. Currently, ACTs are the only recommended antimalarial medicines for uncomplicated malaria in many developing countries like Ghana; hence caution should be taken to ensure its rational use to curtail the emergence of resistance in the near future [8]. It is also important that suggested ways of preventing antimalarial resistance are followed. These include more selective use of drugs and improved prescribing practices and strategies to improve patient adherence. Such interventions must also be continually monitored and assessed [3]. Adherence refers to the extent to which patients follow through decisions about medicine taking [9]. A number of studies have focused on assessing patient adherence to antimalarials and some factors associated with adherence. Adherence levels (based on pill count) are estimated to be between 40% and 70% [10, 11]. Attendance to a particular health center and instructions not to give medication to another person and to return if fever persists are some factors associated with complete adherence. Others are taking the first dose at the health centre, a preference for Artemether Lumefantrine (AL) compared with other antimalarials and patients' knowledge with respect to the ACT dosage regimen [10–12].

The Affordable Medicines Facility for Malaria (AMFM) initiated in Ghana may have led to an expansion of ACTs for the management of malaria as they are easily available and affordable [13]. Measures should therefore be taken to ensure and monitor its rational use to avoid the emergence of resistance. Research data assessing patient related factors that affect adherence to antimalarial medication are scanty. Searching through the literature, only few studies were identified which explored adherence and patients' knowledge on some aspects of malaria and its treatment. There is therefore the need to address this knowledge gap so that health care practitioners may be informed on which facts on malaria and its treatments the patient should be very much aware of when antimalarials are prescribed and dispensed.

*Aim of the Study.* It is to explore patient's knowledge and beliefs on malaria and its treatment and how it affects adherence.

## 2. Methodology

### 2.1. Ethical Considerations

Permission for the research was sought and granted by the Robert Gordon University Ethical Committee and the Committee on Human Research Publications and Ethics, Kwame Nkrumah University of Science and Technology, School of Medical Sciences, and Komfo Anokye Teaching Hospital, Kumasi, Ghana.

### 2.2. Study Area and Sample

The study was conducted in a community pharmacy situated in Lakeside Estate, a suburb of Accra, the capital of Ghana. The community pharmacy setting was chosen because it is one of the common primary health facilities where malaria is managed. In Ghana, ACTs are commonly prescribed by many of the health care personnel working in different health care settings. However physicians practising hospitals and health centres normally prescribe ACTs. Pharmacists practising in the community pharmacy may also prescribe ACTs if a patient presents with signs and symptoms consistent with malaria and it is confirmed using a Rapid Diagnostic Test (RDT) kit. Our inclusion criteria were patients to whom ACTs have been prescribed by any of these health care practitioners and dispensed from the community pharmacy. Patients aged 0–17 years and patients with no contact numbers were excluded. The sample size required was calculated according to the formula *n* = *t* ^2^ × *p*(1 − *p*)/*m* ^2^ (*t* = confidence level at 95%; margin of error at 5%; *p* = estimated prevalence of 30%). From this equation a sample of 277 was obtained. However, the sample was further increased by 5% to account for contingencies. A total of 356 subjects were recruited for the study.

### 2.3. Development and Validation of Questionnaire

A semistructured questionnaire was designed for a face-to-face interview with the patient with reference to the Beliefs and Behaviour Questionnaire (BBQs), knowledge attributes and practices questionnaire (KAP) [14, 15], and literature on the causes, signs, symptoms, and management of uncomplicated malaria. Two community pharmacists and an academic pharmacist face validated the semistructured questionnaire. The questionnaire was further retested to ensure its reliability. There is no standard method for measuring adherence [16]. Methods available such as electronic prescribing and patient self-report both have certain faults, and hence some authors suggest that more than one method for measuring adherence should normally be used [16, 17]. Although certain methods of measuring adherence may be preferred in specific clinical or research settings, a combination of measures maximizes accuracy [17]. This is because it helps to derive the advantages of the two methods used. Hence ideally, one self-report measure and electronic prescribing (Medication Event Monitoring System, MEMS) should be used for the study. Medication Event Monitoring System (MEMS) could have been very appropriate for the study. The medication bottle has a cap containing a microprocessor which records date and time of all the opening. However, the MEMS method cannot be applied to this study although it would have added to the reliability of the study because it is not available in Ghana. In most researches that have studied the adherence to antimalarials with respect to malaria and its treatment, pill count was the only adherence measure used [10–12]. Hence in our study, pill count was used to explore adherence to the ACTs.

### 2.4. Data Collection

Data was collected over a period of five months, from April 1, 2013, to August 31, 2013. Five randomly selected patients fitting into the inclusion criteria were recruited every day from Tuesday to Thursday and follow-ups were from Friday to Monday. Consent was sought before recruitment. On the day of recruitment patients were educated on the causes, signs, symptoms, and prevention of malaria. They were also educated on the name, dosage, side effects and efficacy of the antimalarial dispensed, cautionary measures to observe while taking their antimalarial, and consequences of not completing their antimalarial dosages before antimalarials were dispensed. On the fourth day after the three-day therapy, patients were followed up to assess adherence. Adherence was assessed by pill counting of antimalarial packs. Furthermore, a face-to-face interview with the semistructured questionnaire was used to assess respondents' beliefs on malaria and knowledge on the causes, signs, symptoms, and treatment of malaria. It was the researcher and other trained data collectors who collected the data. The recommended ACTs considered included Artesunate Amodiaquine (AA), Artemether Lumefantrine (AL), and Dihydroartemisinin Piperaquine (DP).

### 2.5. Data Analysis

SPSS version 17 was used for data analysis. To measure the level of adherence to antimalarial medication based on pill count, the number of tablets taken was converted into percentages and defined as follows: 100% fully adherent; 70%–<100% partially adherent; <70% nonadherent [9]. *P* values obtained by comparing patient's knowledge scores and adherence were said to be significant if *P* value was less than 0.05. Cramer's *V* test was used to determine the strength of the association; values of 0–0.30 were considered as weak, 0.31–0.70 as moderate, and 0.71–1.0 as strong. To determine respondents' knowledge score on the causes, symptoms, and prevention of malaria, a scoring system was employed where patients were given a score on each correct answer provided for causes, symptoms, prevention of malaria, with a total of 5 marks being awarded. With regard to respondents' knowledge on treatment of malaria, a scoring system was also used, where respondents were given a mark for each correct answer on the following variables: name of the prescribed ACTs; dosage of the ACTs; precaution to observe while taking the ACTs; any side effects of the ACTs; efficacy of the ACTs; and consequences of untreated malaria.

## 3. Results

Three hundred and fifty-six (356) participants were recruited for the study and 56 were lost to follow-up.

### 3.1. Antimalarial Dispensed and Adherence to Antimalarial Medication

Two hundred and nineteen respondents (73.0%) dispensed AL, 55 (18.3%) AA, and 26 (8.7%) AP (Table 1). Based on the pill count assessment 172 (57.33%) respondents were adherent.

### 3.2. Knowledge on Causes, Signs, Symptoms, and Prevention of Malaria and Its Effects on Adherence

All three hundred (100%) respondents had heard of malaria. Modes of transmission of malaria cited included mosquito bite 296 (98.7%), weather 2 (0.67%), and evil spirit 1 (0.33%). One hundred (57.3%) respondents were aware of headache, body pains, fever, and loss of appetite as common signs and symptoms of malaria. Two hundred and five (68.3%) respondents knew three methods for the prevention of malaria. These were the use of bed nets, mosquito spray, repellents, and mosquito coil. The mean patients knowledge score on causes, signs, symptoms, and prevention of malaria was 3.18 (SD = 0.852) out of 5. The *P* and Cramer's *V* values obtained for the relationship between adherence and patient overall knowledge on the causes, signs, symptoms, and prevention of malaria were chi square: 12.467; df = 8; *P* = 0.132; *V* = 0.144.

### 3.3. Knowledge on Treatment of Malaria and Its Relationship to Adherence

**Figure 1:**
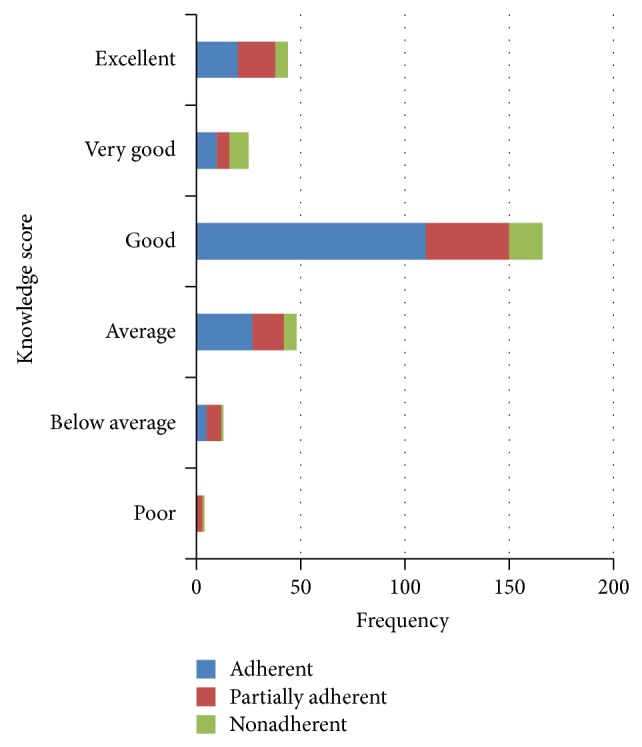
The relationship between adherence and patients' knowledge score on medicines prescribed for antimalarial.

Twenty-seven (56.3%) respondents out of the 48 respondents that had average knowledge on the treatment of malaria were adherent, 15 (31.30%) partially adherent, and 6 (12.50%) nonadherent (Figure 1). Two hundred and seventy (89.4%) respondents agreed that the ACTs were effective. Two hundred and twenty-one respondents (73.2%) indicated that the ACTs did not have side effects, while 79 (26.2%) respondents said the ACTs have side effects (Table 2). Side effects cited included nausea 76 (96.2%), vomiting 78 (98.7%), and dizziness 66 (83.5%). Precautions to observe while taking their antimalarial medication cited by respondents included taking medicine after food 44 (14.7%), finishing all medications 60 (20.0), and taking them as directed 107 (35.7%). Two hundred and seventy-two (90.1%) respondents knew malaria can kill if untreated. The mean patient's knowledge score on treatment of malaria was 4.0867 out of 6 (SD = 1.069). The *P* and Cramer *V*'s values obtained for the relationship between adherence and patients' overall knowledge on the ACTs used for the treatment of malaria was (chi square: 30.354; df = 12; *P* = 0.002; *V* = 0.325).

### 3.4. Beliefs on Malaria and Its Relationship to Adherence

Eighty-four (28.2%) respondents agreed that prayers, instead of medicines taken, healed them from malaria. One hundred and fifty-one (50.3%) respondents felt that prescribers place too much trust on medicines and that if prescribers had more time with patients they would prescribe fewer medicines. The *P* and Cramer's *V* values obtained for the effect of the belief that natural remedies are safer than medicines were 0.000 and 0.345 and those obtained for the belief that “prescribers place too much trust in medicines” on adherence were 0.006 (*v* = 0.570), respectively. The other beliefs of respondents assessed had no effect on adherence to malaria medications (Table 3).

### 3.5. Patients Demographics and Their Relationship to Adherence

One hundred and sixty-three respondents (54%) were males and 137 (45.4%) were females. Nine respondents (3%) had no education, 150 (50%) had tertiary education, and 82 (27.2%) had senior high school education. The chi square value obtained for the relationship between adherence and patients' age was 14.276 (df 8;  *P* = 0.075) while that obtained for the relationship between adherence and educational level of patients was 25.817 (df 12;  *P* = 0.011) (Table 4).

## 4. Discussion

AL was the most frequently antimalarial dispensed. In a similar study in rural Tanzania, Artemether Lumefantrine (AL) was the most common antimalarial dispensed from the drug stores followed by sulfadoxine pyrimethamine, metakelfin, amodiaquine, quinine, and chloroquine [18]. Although AL was the antimalarial frequently dispensed, prescription pattern varied from the results in our study. Currently in Ghana, the ACTs are the only recommended medicines available for uncomplicated malaria in nonpregnant adults and children. Single therapies are not available [8]. AL obviously may have become the drug of choice by most prescribers in Ghana probably because AL and AA are the two antimalarials supported by the AMFM initiative; hence they are readily available and affordable. Secondly, prescribers in Ghana may have preferred AL to AA because in 2005 there were reports of adverse drug reaction to the AA combination which led to the withdrawal of some preparations from the market; hence prescribers currently may be reluctant to prescribe it [19].

Adherence level to the ACTs was 57.3%. In a similar study in rural Kenya, adherence (assessed by pill count) to the ACTs was 42.1% [11]. Percentage level of adherence obtained is slightly higher than that obtained from the research in Kenya. This could be because half (50.0%) of our sample had education up to the tertiary level compared to the study in Kenya which had a high proportion of the sample with education up to the primary level. Considering the various ACTs, adherence to AL was 61.6%. This result is similar to studies in Malawi and two districts (Garissa and Bunyala) in Kenya where adherence to AL was 65% and 64.1%, respectively [10, 12]. Adherence to AA was 45%; the value is slightly lower compared to a study in Sierra Leone where adherence to AA was 57% [20]. The study in Sierra Leone was carried out straightaway after the successful introduction of a new malaria treatment and this may have affected the outcomes of the study. After a new innovation, health professionals renewed from training are highly motivated and are more likely to pay attention to the information given to patients. However, there is the need for interventions to increase the level of adherence to the ACTs by hundred percent because malaria is an acute infectious disease and the pathogens are only killed when the full dose is taken. Subtherapeutic dosages as a result of patients not completing their dosages could lead to drug resistance, and this could alter the progress made towards eradicating malaria in developing countries. The Ghana Malaria Control Board in collaboration with Pharmacy Council Ghana could adopt some interventions that have been studied to enhance adherence to ACTs. These include sending text message reminders to pharmacy staff on the advice to give when dispensing ACTs and text message reminders on adherence to therapy within twelve-hour intervals to patients [21].

The mean knowledge score on the causes, symptoms, and prevention of malaria was 3.18 (SD = 0.852). This can be attributed, to some extent, to the high level of malaria education in the country. Despite very good knowledge on the causes, signs, symptoms, and prevention of malaria, this study also revealed evidence of knowledge gaps about malaria by few respondents comparable to other studies [22, 23]. Considering the *P* and Cramer's *V* values obtained for the relationship between adherence to ACTs and patients' knowledge with respect to causes, signs, symptoms, and prevention of malaria, there was no significant relationship.

The mean knowledge score on treatment of malaria was 4.0867 (SD = 1.069). More than 80.0% of our respondents were of the view that the ACTs were effective and also knew the name of the ACTs dispensed which was encouraging. However, there were some knowledge gaps among respondents. About 73.8% of respondents were of the view that the ACTs were free of side effects. A significant and moderate relationship was established between adherence and patients' knowledge on the dosage of ACT prescribed. These results are consistent with a similar study carried out in Kenya in which patients' knowledge of the dosing regimen was associated with high adherence levels [12]. This underlines the need for initiatives in community pharmacies to enhance counselling on the dosage, effectiveness, and side effects of the ACTs when they are dispensed. Education could be offered at the point of care and educational materials could accompany medicines dispensed. For patients who are illiterates, pictograms could be used to ensure they understand the dosing regimen and consequences of not completing their medicines. Furthermore, text messages could be sent to patients educating them on the side effects of the ACTs and some precautionary measures to be observed so as to minimize these side effects. There is also the need for continuous education for all pharmacy staff to sharpen their skills with regard to the pharmaceutical care offered to patients with malaria, because the community pharmacy is the first port of call of some people with malaria.

Human beings have different beliefs and this can have a significant impact on their behaviour. Thirty-four respondents who agreed that prayers instead of medication healed their malaria were partially adherent. These results are consistent with others that highlight the essential role that spiritual beliefs and practices play in understanding disease in the African culture and the belief that God is ultimately responsible for physical and spiritual health [24]. Significant and moderate relationship was established between adherence and some beliefs. Although people's beliefs cannot be changed easily, there is the need to consider the belief of patients when care is offered as this can affect adherence to the ACTs. For instance, the belief that “prescribers place too much trust in medicines” could be addressed if community pharmacists and staff use a concordance approach, where the patient's opinions and beliefs are considered when care is offered. In addition, the belief that “natural remedies are safer than medicines” and “people who take medicines can stop their treatment for a while every now and then” could be addressed when this approach is used.

The *P* value obtained for the relationship between adherence to ACTs and respondent's educational level was 0.011 (Table 4). In a similar study in Uganda where pill count was used to assess adherence, educational level was a factor influencing adherence to ACTs but age was not associated with adherence [25]. However, in a previous study in Kenya both age and educational background of respondent were found to be a significant predictor of adherence [11, 12]. There was no significant relationship between adherence and gender of patients. A limitation to the study was that the research was a nonrandomised single site study. Although the participants in this study may reflect the sociodemographic characteristics of malaria for patients in some parts of Accra, they may not be representative of characteristics in other geographical areas of Ghana. These limitations however did not impact the results negatively.

## 5. Conclusion

Patient adherence level to the ACTs is moderate. In the process of dispensing antimalarials, counseling should focus more on the consequences of not completing antimalarial doses, dosage, efficacy, and side effects of the antimalarials and less on the causes, signs, symptoms, and prevention of malaria. Furthermore patients' beliefs and opinions should consider when antimalarials are prescribed.

## Figures and Tables

**Table 1:** Adherence to ACTs.

ACTs	Adherent	Partially adherent	Nonadherent	Total
Artemether/Lumefantrine (AL)	135 (61.6)	67 (30.6)	17 (7.8)	219
Artesunate/Amodiaquine (AA)	25 (45.4)	14 (25.4)	16 (29.1)	55
Artesunate/piperazine (AP)	12 (46.1)	8 (30.7)	6 (23.0)	26
				**300**

**Table 2:** Knowledge on treatment of malaria and its relationship to adherence.

Respondents awareness	*N*	Adh	Par Adh	Non Adh	*P* value	*V*
The name ACTs recommended for the management of malaria	264 (88%)	157	73	34	0.910	0.126
Dosage of ACTs recommended for the management of malaria	252 (86%)	160	65	40	0.007	0.457
Efficacy of antimalarial	269 (89%)	162	76	31	0.009	0.377
Side effects of antimalarial	79 (26%)	31	30	18	0.000	0.403
Cautionary and advisory measures to observe while taking antimalarial	268 (89%)	162	74	32	0.456	0.110
The consequences of not completing the doses of antimalarial dispensed	272 (90%)	161	77	34	0.001	0.309

^*^Adh: adherent; ^*^Par Adh: partially adherent; ^*^Non Adh: nonadherent; *V*: Cramer's *V*.

**Table 3:** Relationship between adherence and beliefs on malaria and its treatment.

Belief	Response	Adherent	Partially adherent	Nonadherent	*P* value	*V*
Prayers instead of medicine that I have taken healed me	Agree/strongly agree	41	34	9		
	Uncertain	28	17	12	0.464	0.072
	Disagree/strongly disagree	103	38	18		
Natural remedies are safer than medicines	Agree/strongly agree	40	29	20		
	Uncertain	36	26	14	0.000	0.345
	Disagree/strongly disagree	96	34	5		
People who take medicines can stop their treatment for a while every now and again	Agree/strongly agree	10	10	10		
	Uncertain	10	5	3	0.458	0.458
	Disagree/strongly disagree	152	74	26		
Most medicines including malaria medicines are addictive	Agree/strongly agree	15	13	6		
	Uncertain	46	24	11	0.554	0.061
	Disagree/strongly disagree	111	52	22		
Medicines do more harm than good	Agree/strongly agree	41	25	15		
	Uncertain	24	11	4	0.465	0.054
	Disagree/strongly disagree	107	53	20		
All medicines are poisons	Agree/strongly agree	37	19	5		
	Uncertain	16	15	9	0.498	0.080
	Disagree/strongly disagree	119	55	25		
Prescribers place too much trust on medicines	Agree/strongly agree	73	54	24	0.006	0.570
	Uncertain	25	4	1		
	Disagree/strongly disagree	74	31	14		
If prescribers had more time with patients they will prescribe fewer medicines	Agree/strongly agree	73	54	24		
	Uncertain	25	4	1	0.423	0.059
	Disagree/strongly disagree	74	31	14		

**Table 4:** The relationship between adherence level and demographic characteristics.

Demographics		Adherent	Partially adherent	Nonadherent	*P* value (*V*)
Age	18–25	61	34	24	0.75 (0.154)
	26–35	50	20	5	
	36–45	24	16	3	
	46–55	21	15	4	
	56 and above	16	4	3	

Gender	Male	93	49	21	0.986 (0.010)
	Female	79	40	18	

Educational level	No education	3	2	4	0.011 (0.207)
	Primary	5	6	0	
	Middle school	5	6	0	
	JHS	16	15	6	
	SHS	48	25	9	
	Tertiary	95	35	20	

## Abbreviations

AA: Artesunate Amodiaquine
ACT: Artemisinin based combination therapy
AL: Artemether Lumefantrine
AMFM: Affordable Medicines Facility for Malaria
BBQ: Beliefs and Behavior Questionnaire
CQ: Chloroquine
DP: Dihydroartemisinin Piperaquine
KAP: Knowledge attributes and practices questionnaire
MEMS: Medication Event Monitoring System
RDT: Rapid Diagnostic Test
SPSS: Statistical Package for Social Science
WHO: World Health Organization.

## References

[1] WHO http://www.who.int/mediacentre/factsheets/fs094/en/.

[2] Ghana Health Service http://ghanahealthservice.org/ghs-subcategory.php?cid=4&scid=41.

[3] Bloland P. B. Drug resistance in malaria. http://www.who.int/csr/resources/publications/drugresist/malaria.pdf.

[4] WHO Roll Back Malaria Partnerships: Facts on ACTs (Artemisinin Based Combination Therapy). http://www.rollbackmalaria.org/psm/acts.html.

[5] Noedl H., Se Y., Schaecher K., Smith B. L., Socheat D., Fukuda M. M. (2008). Evidence of artemisinin-resistant malaria in Western Cambodia. *The New England Journal of Medicine*.

[6] Dondorp A. M., Nosten F., Yi P. (2009). Artemisinin resistance in *Plasmodium falciparum* malaria. *The New England Journal of Medicine*.

[7] WHO World Malaria Report. http://www.who.int/malaria/world_malaria_report_2011/9789241564403_eng.pdf.

[8] Ministry of Health (2013). *Antimalarial Drug Policy*.

[9] de las Cuevas C. (2011). Towards a clarification of terminology in medicine taking behavior: compliance, adherence and concordance are related although different terms with different uses. *Current Clinical Pharmacology*.

[10] MacE K. E., Mwandama D., Jafali J. (2011). Adherence to treatment with artemether-lumefantrine for uncomplicated Malaria in Rural Malawi. *Clinical Infectious Diseases*.

[11] Onyango E. O., Ayodo G., Watsierah C. A. (2012). Factors associated with non-adherence to Artemisinin-based combination therapy (ACT) to malaria in a rural population from holoendemic region of western Kenya. *BMC Infectious Diseases*.

[12] Lawford H., Zurovac D., O'Reilly L. (2011). Adherence to prescribed artemisinin-based combination therapy in Garissa and Bunyala districts, Kenya. *Malaria Journal*.

[13] Ghana Health Service (GHS) (2011). *National Malaria Control Programme Annual Report, 2010*.

[14] George J., Mackinnon A., Kong D. C. M., Stewart K. (2006). Development and validation of the Beliefs and Behaviour Questionnaire (BBQ). *Patient Education and Counseling*.

[15] Hlongwana K. W., Mabaso M. L. H., Kunene S., Govender D., Maharaj R. (2009). Community knowledge, attitudes and practices (KAP) on malaria in Swaziland: a country earmarked for malaria elimination. *Malaria Journal*.

[16] Osterberg L., Blaschke T. (2005). Adherence to medication. *The New England Journal of Medicine*.

[17] Horne R., Weinman J., Barber N. (2005). *Concordance, Adherence and Compliance in Medicine Taking*.

[18] Mazigo H. D., Obasy E., Mauka W. (2010). Knowledge, attitudes, and practices about malaria and its control in rural Northwest Tanzania. *Malaria Research and Treatment*.

[19] Ghana Web Ministry orders the withdrawal of new malaria drug. http://mobile.ghanaweb.com/GhanaHomePage/NewsArchive/artikel.php?ID=96406.

[20] Gerstl S., Dunkley S., Mukhtar A., Baker S., Maikere J. (2010). Successful introduction of artesunate combination therapy is not enough to fight malaria: results from an adherence study in Sierra Leone. *Transactions of the Royal Society of Tropical Medicine and Hygiene*.

[21] Goodman C., Maloney K. Enhancing adherence to ACTs purchased from drug shops: results from four intervention studies. http://www.actconsortium.org/data/files/Symposia_MIM/Symposium_4_Enhancing_adherence_to_ACTs_purchased_from_drug_shops.pdf.

[22] Abate A., Degarege A., Erko B. (2013). Community knowledge, attitude and practice about malaria in a low endemic setting of Shewa Robit Town, northeastern Ethiopia. *BMC Public Health*.

[23] Kinung'Hi S. M., Mashauri F., Mwanga J. R. (2010). Knowledge, attitudes and practices about malaria among communities: comparing epidemic and non-epidemic prone communities of Muleba district, North-Western Tanzania. *BMC Public Health*.

[24] Kemppainen J., Kim-Godwin Y. S., Reynolds N. R., Spencer V. S. (2008). Beliefs about HIV disease and medication adherence in persons living with HIV/AIDS in rural southeastern North Carolina. *Journal of the Association of Nurses in AIDS Care*.

[25] Fogg C., Bajunirwe F., Piola P. (2004). Adherence to a six-dose regimen of artemether-lumefantrine for treatment of uncomplicated Plasmodium falciparum malaria in Uganda. *American Journal of Tropical Medicine and Hygiene*.

